# Characterization and antimicrobial potential of bacteriocin-producing lactic acid bacteria isolated from the gut of *Blattella germanica*

**DOI:** 10.1128/spectrum.01203-25

**Published:** 2025-09-23

**Authors:** Yuqi Wei, Fangmin Chen, Wanting Xia, Jinyue Song, Jinyan Liang, Xinyao Yang

**Affiliations:** 1 Liaoning Province Key Laboratory of Urban Integrated Pest Management and Ecological Security, College of Life Science and Bioengineering, Shenyang University 47824, Shenyang, Liaoning, China; 2 Key Laboratory of Ecological Restoration of Regional Contaminated Environment, Ministry of Education, Shenyang University 47824, Shenyang, Liaoning, China; University of Dundee, Dundee, United Kingdom

**Keywords:** *Blattella germanica*, gut microbiota, bacteriocin, lactic acid bacteria, antimicrobial activity

## Abstract

The gut microbiota of insects plays a crucial role in host nutrition, immune regulation, and disease resistance. As a globally distributed pest, *Blattella germanica* has adapted to diverse environments and maintains a highly varied diet, making its gut microbiota a largely untapped reservoir of antimicrobial compounds. This study systematically investigated the culturable gut microbiota of *Blattella germanica*, leading to the isolation of 30 bacterial strains. Morphological characterization and 16S rRNA gene sequence analysis classified these strains into two phyla and eight genera. Among them, six lactic acid bacteria strains were found to produce bacteriocins. Notably, strain X24 exhibited the strongest antibacterial activity, particularly against *Escherichia coli*. The ethyl acetate-based extraction increased its activity by 39.50%, yielding a concentration of approximately 612.74 µg/mL. Bacteriocin X24 demonstrated a broad antimicrobial spectrum, encompassing both Gram-positive and Gram-negative bacteria as well as fungi. It showed the strongest activity against *Escherichia coli* (MIC = 37.5 µg/mL), moderate activity against *Staphylococcus aureus* and *Bacillus subtilis* (MIC = 75 µg/mL), and relatively weak activity against *Pseudomonas aeruginosa*, both standard and antibiotic-resistant *Salmonella typhimurium*, *Serratia marcescens*, and *Enterococcus faecalis* (MIC = 150 µg/mL) and *Saccharomyces cerevisiae* (MIC = 300 µg/mL). The bacteriocin demonstrated good thermostability, maintained stable antibacterial activity under neutral to slightly acidic (pH 5–7) conditions, and exhibited sensitivity to proteases. This study shows that bacteriocin-producing bacteria from German cockroach guts are a new source of bacteriocins. These could be used in food preservation, biocontrol, and reduction of antibiotic use. The work expands known bacteriocin producers and reveals insect-derived antimicrobial peptides as promising alternatives against drug-resistant pathogens.

**IMPORTANCE:**

The rise of antibiotic-resistant pathogens has intensified the search for novel antimicrobial agents. This study explores the gut microbiota of *Blattella germanica*, a highly adaptable pest, as an untapped source of bacteriocin-producing lactic acid bacteria (LAB). We identified six LAB strains with potent antimicrobial activity by producing bacteriocin, particularly X24, which exhibits broad-spectrum inhibition against Gram-positive (G^+^) and Gram-negative (G^−^) pathogens, including fungi. The bacteriocin demonstrated remarkable stability under high temperatures and varying pH levels, making it a promising candidate for food preservation and biomedical applications. By uncovering the antimicrobial potential of insect-derived LAB, this research expands the diversity of bacteriocin sources and offers a sustainable strategy to combat antimicrobial resistance. Our findings highlight the ecological and biotechnological value of pest-associated microbes, paving the way for innovative alternatives to conventional antibiotics.

## INTRODUCTION

The gut microbiota of insects plays a pivotal role in host nutrient metabolism, immune regulation, and disease resistance, making it a central focus in ecological and microbiological research (1). Studies have shown that the gut microbiota of *Apis mellifera*, *Aedes aegypti*, and *Drosophila melanogaster* significantly influences host growth, development, and defense against pathogen (2–4). The German cockroach (*Blattella germanica*), a globally distributed urban pest, has garnered increasing attention due to the remarkable diversity of its gut microbiota and its critical role in insect physiology and ecology (5). Research indicates that the gut microbiota of the *Blattella germanica* is predominantly composed of *Bacteroidetes*, *Firmicutes*, *Proteobacteria*, and a small proportion of *Actinobacteria* (6). These microbes synergistically participate in the breakdown of carbohydrates, protein metabolism, and lipid synthesis, thereby providing the host with essential energy and necessary metabolic substrates (7). In addition to its roles in digestion and metabolism, the gut microbiota of *Blattella germanica* also significantly contributes to immune regulation and pathogen defense (8). Furthermore, certain gut bacteria have been shown to perform specialized functions, such as pesticide degradation, heavy metal tolerance, and antibiotic resistance, enabling *Blattella germanica* to withstand environmental stressors (9, 10).

Lactic acid bacteria (LAB), as key symbiotic organisms in the insect gut, play a vital role in various physiological processes. There is growing interest in the ecological functions and metabolic products of LAB (11). Bacteriocins, ribosomally synthesized antimicrobial peptides produced by LAB, effectively inhibit Gram-positive (G^+^) and some Gram-negative (G^−^) pathogens (12). These bacteriocins exhibit promising applications in medicine, animal husbandry, and the food industry (13–15). For instance, bacteriocins produced by *Lactobacillus fermentum* isolated from goat milk have been used to extend the shelf life of bananas with remarkable results (16). Antimicrobial peptides produced by *Lactobacillus* strains isolated from human breast milk have been shown to alleviate inflammatory bowel disease (IBD), reduce intestinal infections, and improve gut microbiota balance (17). Similarly, two types of LAB in the intestines of piglets have been found to secrete the bacteriocin gassericin A, which significantly alleviates symptoms of diarrhea (18). Studies have also identified LAB with potent antibacterial activity from the gut of silkworms, honeybees, and ants (19). However, investigating the diversity and bacteriocin production capacity of LAB in the gut of *Blattella germanica* remains highly necessary.

The objectives of this study are as follows: to isolate and identify the bacteria from the gut microbiota of *Blattella germanica*, to screen for strains with high bacteriocin-producing potential, and to evaluate their antimicrobial properties. This research aims to provide both theoretical insights and practical evidence for the development of novel antimicrobial agents. Moreover, the study proposes an innovative approach for discovering strains with unique antimicrobial properties by exploring bacteriocin-producing LAB from unconventional ecological niches, specifically the gut ecosystem of pest insects.

## MATERIALS AND METHODS

### Isolation and characterization of culturable bacteria

In this study, 35 *Blattella germanica* were surface-sterilized and subsequently dissected under aseptic conditions. The entire gut of each cockroach was homogenized in 1.5 mL of 0.85% (m/v) NaCl solution. The gut sample homogenate was subjected to tenfold serial dilutions, generating six consecutive dilution gradients from 10^−2^ to 10^−7^. From each dilution, 100 µL aliquots were aseptically plated in triplicate onto both 1/3 Tryptone Soy Agar (TSA) and De Man, Rogosa, and Sharpe (MRS) agar media. After inverted incubation at 37°C in a constant temperature incubator for 24–48 h, observations were conducted. Distinct single colonies with unique morphological characteristics were carefully selected and underwent sequential isolation and purification procedures. Strain purity was verified through morphological assessment and Gram staining, which were examined using a Leica stereomicroscope. The purified strains were assigned unique identification codes and preserved in 50% (vol/vol) glycerol suspensions at −80°C for long-term storage and future experimental use.

Bacterial identification was performed through morphological characterization and 16S rRNA gene sequence analysis. Genomic DNA was extracted using a commercial bacterial DNA isolation kit (Beijing Dingguo Changsheng Biotechnology Co., Ltd.). The 16S rRNA gene was amplified using universal bacterial primers 27F (5′-AGAGTTTGATCCTGGCTCAG-3′) and 1492R (5′-TACGGCTACCTTGTTACGACTT-3′) with the following PCR protocol: initial denaturation at 95°C for 3 min; 35 cycles of 95°C for 30 s, 55°C for 40 s, and 72°C for 40 s; followed by final extension at 72°C for 5 min. PCR reactions (50 µL) were performed in a gradient thermal cycler (Agilent Technologies Inc.), with template-free negative controls included in each run. Amplification products were separated on 1% agarose gels, visualized using a Bio-Rad gel documentation system, and purified for bidirectional sequencing (Sango Biotech Co., Ltd., Shanghai, China). Sequence assembly and analysis were conducted using BLAST against the NCBI database, with >99% identity indicating conspecific strains and 95–99% identity suggesting congeneric classification (20).

### Identification of LAB strains

Putative LAB strains were initially screened using a two-step biochemical protocol (21): catalase activity testing followed by calcium carbonate dissolution assessment. G^+^ bacteria isolates were first cultured on MRS agar. Catalase activity was evaluated by applying 3.0% (vol/vol) hydrogen peroxide to bacterial smears, with bubble formation indicating positive results. Catalase-negative isolates were then cultured on MRS agar containing 0.7% (wt/vol) calcium carbonate and incubated at 37°C for 24 h. Clear zones surrounding colonies indicated positive calcium carbonate dissolution. G^+^ bacteria, catalase-negative isolates demonstrating calcium carbonate dissolution were presumptively classified as LAB.

### Antibacterial activity of LAB and identification of its antibacterial substance

Antibacterial activity of LAB isolates was evaluated using the Oxford cup method (22, 23). Previous studies indicate that LAB antimicrobial activity may result from organic acids, hydrogen peroxide, or bacteriocins (24). The fermentation broth was cultured to the logarithmic growth phase (37°C), centrifuged (10,000 × *g*, 15 min, 4°C), and filtered through a 0.22 µm membrane to obtain the cell-free supernatant (CFS). CFS of LAB strains with antimicrobial activity was treated to eliminate the effects of organic acid and hydrogen peroxide: 5 mL supernatant was mixed with catalase (5 mg/mL) and adjusted to pH 6.0 using 1 mol/L NaOH before activity assessment against *Escherichia coli* and *Staphylococcus aureus*. Untreated CFS was used to determine bacteriocin activity against the same indicator strain as control.

To determine whether the antibacterial substance possesses proteinaceous characteristics, it was done by mixing 5 mL of the CFS with an equal volume of proteinase K and trypsin mixture (5 mg/mL, 1:1) under their optimum pH 7.4 and incubated at 37°C for 2 h. Enzymatic activity was terminated by boiling for 3 min. Untreated CFS was used to determine bacteriocin activity against *Escherichia coli* as the control. The inhibition zone diameter was measured using the Oxford cup method in all cases.

### Bacteriocin extraction and quantification

Bacteriocins were extracted using an ethyl acetate-based extraction method following established protocols (25). LAB cultures were grown in MRS broth at 37°C with agitation (170 rpm) to logarithmic phase. CFS were prepared by centrifugation (10,000 × *g*, 15 min, 4°C) followed by membrane filtration (0.22 µm). CFS was mixed with ethyl acetate (1:1, vol/vol) and extracted for 2 h. The organic phase was concentrated by rotary evaporation, and bacteriocin content was quantified using a BCA protein assay kit (Beijing Dingguo Changsheng Biotechnology Co., Ltd.). Bacteriocin activity was assessed against logarithmic-phase *Escherichia coli* using the Oxford cup method. After solvent removal, 100 µL of bacteriocin-enriched supernatant (EA extract) was loaded into Oxford cups, using untreated CFS as control. The inhibition zone diameter was measured using the Oxford cup method on Luria-Bertani (LB) agar in all cases.

### Antimicrobial spectrum analysis

The antimicrobial spectrum was evaluated against a diverse panel of indicator microorganisms, including G^+^ bacteria (*Pseudomonas aeruginosa* CMCCB(B)10104, *Escherichia coli* CMCCB(B)44102, standard *Salmonella typhimurium* CMCCB(B)50115, antibiotic-resistant *Salmonella typhimurium*, and *Serratia marcescens*), G^−^ bacteria (*Staphylococcus aureus* CMCCB(B)26003, *Enterococcus faecalis*, and *Bacillus subtilis* ATCC6633), and fungi (*Saccharomyces cerevisiae* ATCC9763). Indicator strains without a registered preservation number were isolated and identified in our laboratory. The same strains of *Escherichia coli* and *Staphylococcus aureus* were used for all other experiments in this study. Bacterial test strains were grown on 2% (wt/vol) LB agar, while fungi test strains were grown on 2% (wt/vol) YPD agar. The inhibition zones were quantitatively measured, and resistance patterns were statistically analyzed to evaluate the efficacy of bacteriocins.

### Assessment of minimum inhibitory concentration (MIC)

In order to determine the MIC value of bacteriocin X24 against test bacterial strains, a broth dilution method was employed (26). Test strains were identical to those described in section “Antimicrobial spectrum analysis.” Indicators were cultured in LB broth or YPD medium at 37°C for 12 h, centrifuged, and the supernatant discarded. The pellet was resuspended in physiological saline (0.85% NaCl) to adjust the optical density at 600 nm (OD_600_) to 0.35. One hundred microliters of twofold serially diluted bacteriocin solutions (300.00–2.34 µg/mL) were dispensed into a 96-well plate (300 µL total volume/well). Each bacterial suspension was diluted 100-fold in fresh medium, and 100 µL was added to bacteriocin-containing wells, yielding a final reaction volume of 200 µL. Controls contained 100 µL saline instead of bacteriocin. After a 24-hour incubation at 37°C, OD_600_ was measured by a microplate reader (infinite 200 PRO, TECAN, Switzerland). MIC was defined as the lowest bacteriocin concentration with OD_600_ statistically significant difference to control (*P* < 0.05).

### Evaluation of bacteriocin stability

To assess thermal stability, 2 mL of EA extract was incubated in a water bath at 37°C, 40°C, 60°C, 80°C, and 100°C for 20 min. The antimicrobial activity of the treated samples was then measured. For pH stability assessment, 1 mL of bacteriocin was dissolved in phosphate-buffered saline (PBS) adjusted to pH values ranging from 2.0 to 8.0 in 1-unit increments using 1 M NaOH or HCl, and incubated at 37°C for 24 h. To determine enzyme stability, 2 mL of bacteriocin was incubated with catalase, trypsin, or proteinase K at 37°C for 30 min, maintaining a constant volume. The enzymatic reactions were terminated by boiling for 3 min, with untreated samples used as controls. In all experiments, antimicrobial activity was assessed using the Oxford cup agar diffusion method, with *Escherichia coli* as the indicator strain.

Bacteriocin stability was evaluated under various conditions. Thermal stability was assessed by incubating 2 mL bacteriocin at 37°C, 40°C, 60°C, 80°C, and 100°C (20 min) in a water bath. The pH stability was determined by dissolving 1 mL bacteriocin in PBS (pH 2.0–8.0, adjusted with 1 M NaOH/HCl) and incubating at 37°C for 24 h.

To evaluate enzyme stability, 2 mL aliquots of bacteriocin were individually treated with trypsin, catalase, and proteinase K at 37°C for 30 min. The enzymatic reactions were subsequently terminated by boiling for 3 min. Parallel untreated samples were used as controls. Antimicrobial activity was systematically assessed against *Escherichia coli* using the standardized Oxford cup method. The inhibition zone diameter was measured using the Oxford cup method on LB agar in all cases.

### Statistical analysis

All experimental data are expressed as mean ± SEM of triplicate measurements. Statistical analysis was performed using GraphPad Prism software (v10.1.2), and one-way ANOVA was employed for multiple group comparisons.

## RESULTS

### Isolation and identification of culturable bacteria: the *Blattella germanica* gut is rich in microbial resources

**Fig 1:**
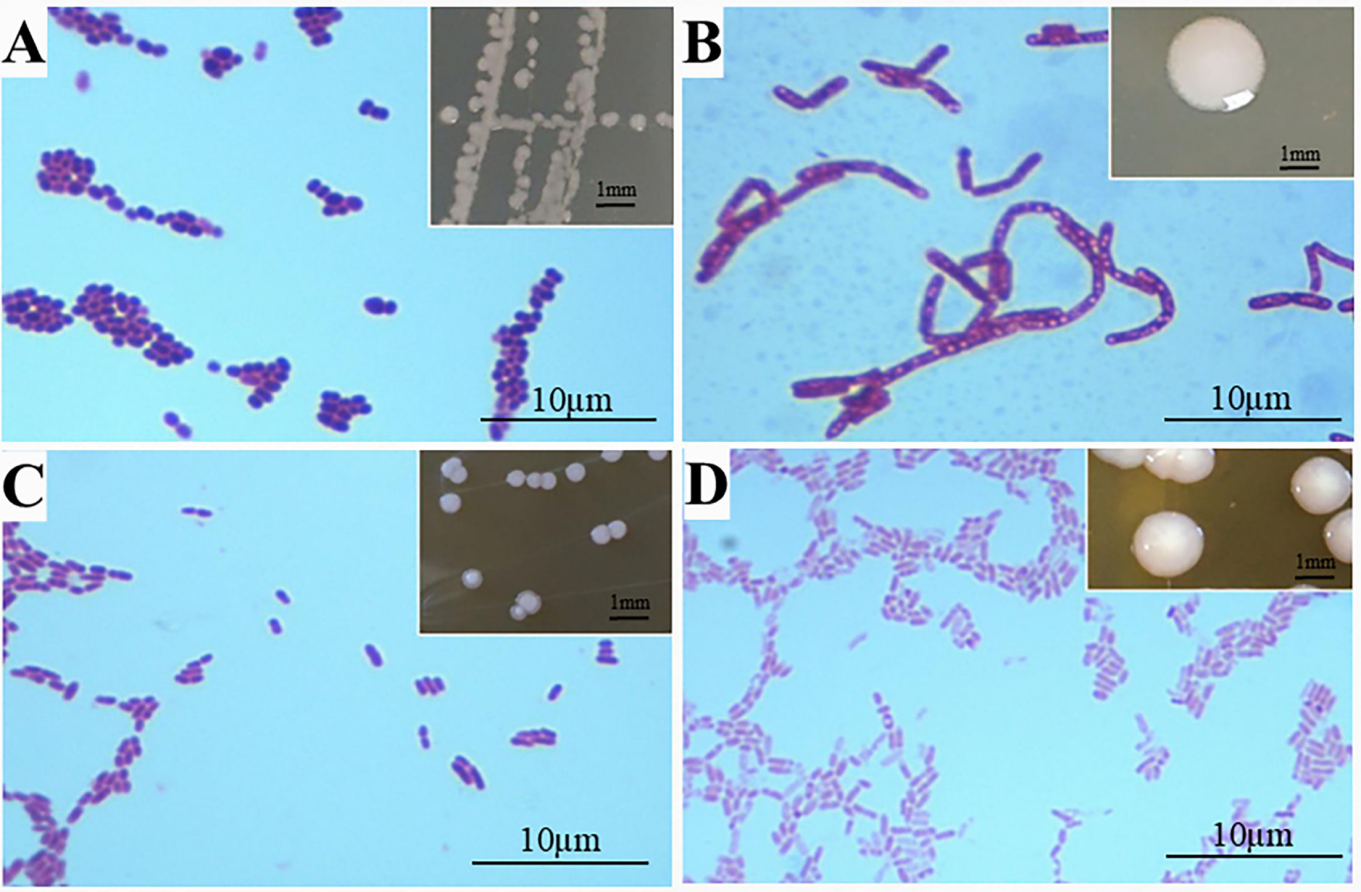
Colony morphology and Gram staining results of representative strains. The upper right panels of (**A–D**) show colony morphology. (**A**) Strain X1, G^+^; (**B**) Strain X8, G^+^; (**C**) Strain X24, G^+^; (**D**) Strain X22, G^−^.

Thirty strains were obtained from the gut of *Blattella germanica*, labeled as X1 to X30. Among them, eight strains (X5, X9, X20, X21, X22, X26, X28, and X30) were G^−^ bacteria, while the remaining 22 strains were G^+^ bacteria. The colony morphology and Gram staining results of representative strains are shown in Fig. 1.

The 16S rRNA gene sequences of strains X1 to X30 have been submitted to the NCBI GenBank database, with the accession numbers as follows: PV243577–PV243606. Combining morphological and molecular biological approaches, the 30 strains were classified into eight genera within two phyla. The cultivable gut microbiota of *Blattella germanica* was dominated by Firmicutes (73.3%), particularly *Bacillus* and *Enterococcus* spp. The Firmicutes phylum comprised *Enterococcus* (eight strains), *Bacillus* (eight strains), *Priestia* (one strain), *Mammaliicoccus* (two strains), and *Staphylococcus* (three strains), while the Proteobacteria phylum included *Enterobacter* (six strains), *Limnobaculum* (one strain), and *Raoultella* (one strain). Notably, strains X5 and X27 showed low similarity to their type strains (94.43% and 91.22%, respectively), suggesting potential novel taxa that warrant genome-based validation. Detailed taxonomic information is provided in Table 1.

**TABLE 1:** Classification of culturable bacterial strains isolated from the gut of *Blattella germanica*

Strain no.	Reference strain	Per. ident	Bacterial genera	Phylum
X8	*Bacillus aryabhattai*	99.65%	*Bacillus*	Firmicutes
X6, X23	*Bacillus cereus*	99.51%		
X10	*Bacillus licheniformis*	99.65%		
X15, X18	*Bacillus safensis*	99.86%		
X29	*Bacillus aerius*	99.79%		
X11	*Bacillus subtilis*	99.30%		
X14	*Priestia flexa*	99.16%	*Priestia*	
X1, X4, X7, X24, X25	*Enterococcus malodoratus*	99.38%	*Enterococcus*	
X12	*Enterococcus canis*	97.87%		
X2	*Enterococcus mundtii*	99.45%		
X27	*Enterococcus avium*	91.22%		
X3, X13	*Mammaliicoccus sciuri*	99.51%	*Mammaliicoccus*	
X16	*Staphylococcus capitis*	99.79%	*Staphylococcus*	
X17, X19	*Staphylococcus haemolyticus*	99.23%		
X28, X30	*Enterobacter bugandensis*	97.88%	Enterobacter	Proteobacteria
X9, X21, X22, X26	*Enterobacter ludwigii*	99.50%		
X5	*Limnobaculum parvum*	94.43%	*Limnobaculum*	
X20	*Raoultella ornithinolytica*	99.37%	*Raoultella*	

The 16S rRNA gene sequence of representative strain of each species was selected to construct phylogenetic tree for phylogenetic analysis (Fig. S1), which confirmed the results of the taxonomic identification.

### Screening and antibacterial activity of bacteriocin-producing LAB

**Fig 2:**
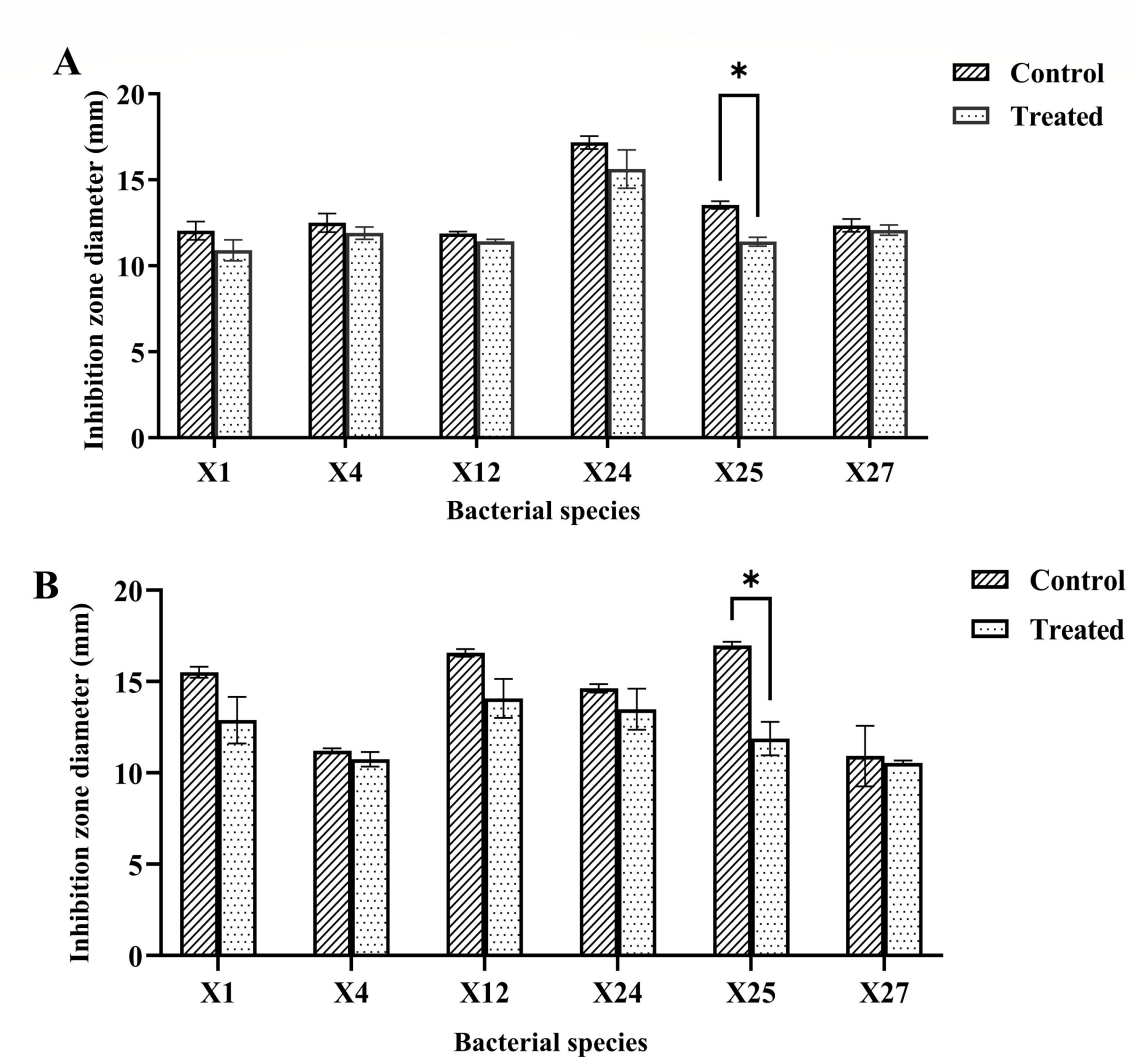
Antibacterial activity of LAB strains before and after the removal of organic acids and hydrogen peroxide. *P < 0.05. (**A**) *Escherichia coli* as indicator. (**B**) *Staphylococcus aureus* as indicator. Control: fermented supernatant. Treated: with catalase and NaOH treated fermented supernatant.

Six LAB strains (X1, X4, X12, X24, X25, and X27) were selected from the initial 30 isolates, consisting of *Enterococcus* sp. The six LAB strains demonstrated varying levels of antibacterial activity against *Escherichia coli* and *Staphylococcus aureu*s. The antibacterial activity of LAB strains before and after the removal of organic acids and hydrogen peroxide from their production is shown in Fig. 2.

The antibacterial assay demonstrated that the six lactic acid bacteria strains exhibited strong inhibitory effects against both *Escherichia coli* and *Staphylococcus aureus*. It demonstrates that strain X24 exhibited the strongest inhibitory effect against *Escherichia coli*, with an inhibition zone diameter of 17.17 ± 0.31 mm (Fig. 2A), while strain X25 showed the best antibacterial activity against *Staphylococcus aureus*, with an inhibition zone diameter of 16.08 ± 0.19 mm (Fig. 2B). The antibacterial activity of strain X25 against both indicator strains underwent significant changes after treatment with hydrogen peroxide and sodium hydroxide (*P* < 0.05). In contrast, the other strains showed no significant changes in antibacterial activity against the two indicator strains (*P* > 0.05), further indicating that their antibacterial effects are unlikely to be mediated by organic acids or hydrogen peroxide. To verify the antimicrobial substances, the fermented supernatant was treated with trypsin and proteinase K, after which no antibacterial activity against *Escherichia coli* was detected (Fig. S2). Therefore, it can be inferred that the antimicrobial activity of LAB is attributed to the production of antimicrobial proteins, specifically bacteriocin. Strain X24 was selected for further investigation due to its superior antimicrobial activity.

### Bacteriocin antimicrobial activity and content analysis

**Fig 3:**
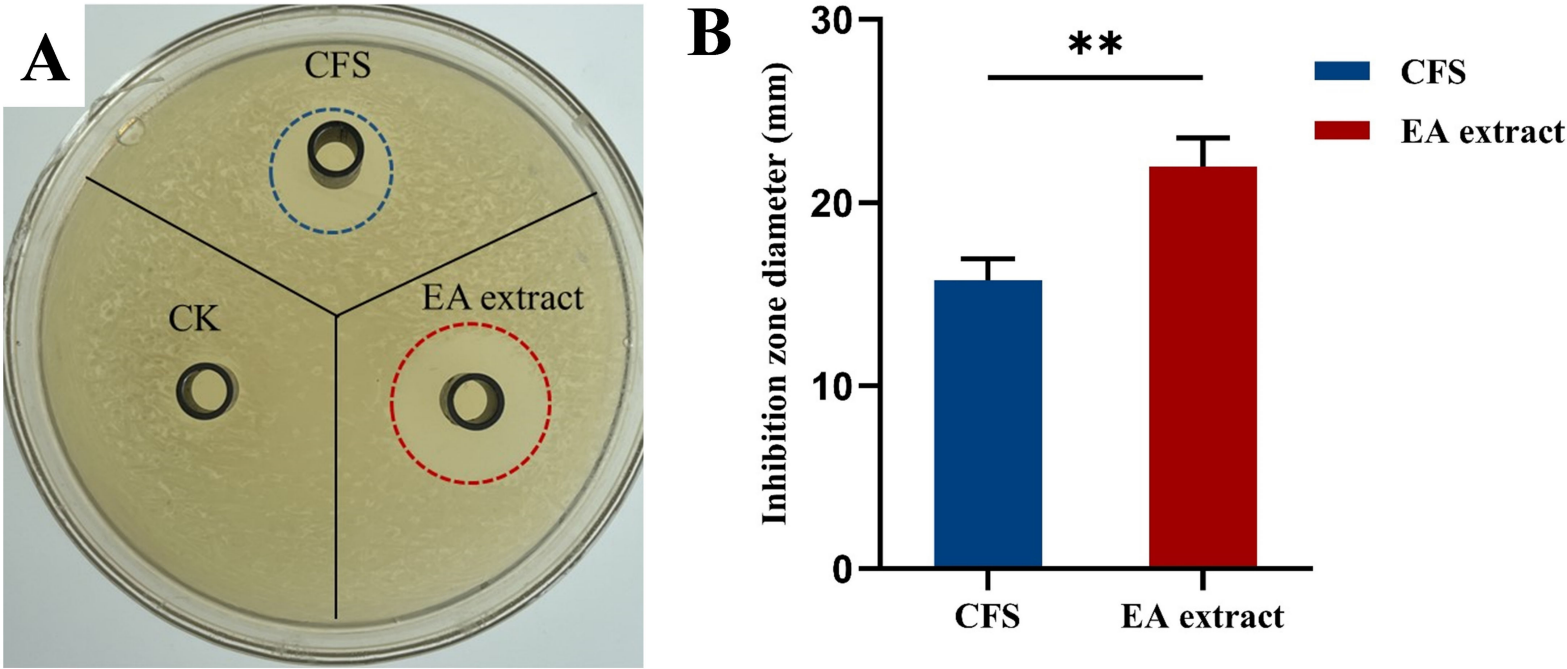
Antimicrobial activity against *Escherichia coli* of CFS and EA extract of strain X24. CFS: Cell-free supernatants. EA extract: CFS with ethyl acetate extraction. (**A**) Image of inhibition zones in the agar plate; blue and red dashed circles indicate the zones of inhibition. CK: sterile water as control. (**B**) Bar chart of statistical analysis; **P < 0.01.

The antimicrobial activity of CFS and that of the ethyl acetate extraction (EA extract) of strain X24 is determined as shown in Fig. 3.

As shown in Fig. 3, the antibacterial activity of CFS is significantly enhanced with ethyl acetate extraction, with the inhibition zone diameter increasing from 15.75 ± 0.95 mm to 21.97 ± 1.27 mm, representing a 39.52% improvement. The bacteriocin concentration of EA extract was 612.74 µg/mL, as determined using a BCA protein assay kit. Subsequent experiments were conducted using ethyl acetate extracts (EA extract) of strain X24 and named bacteriocin X24.

### Antimicrobial spectrum of bacteriocin X24

**Fig 4:**
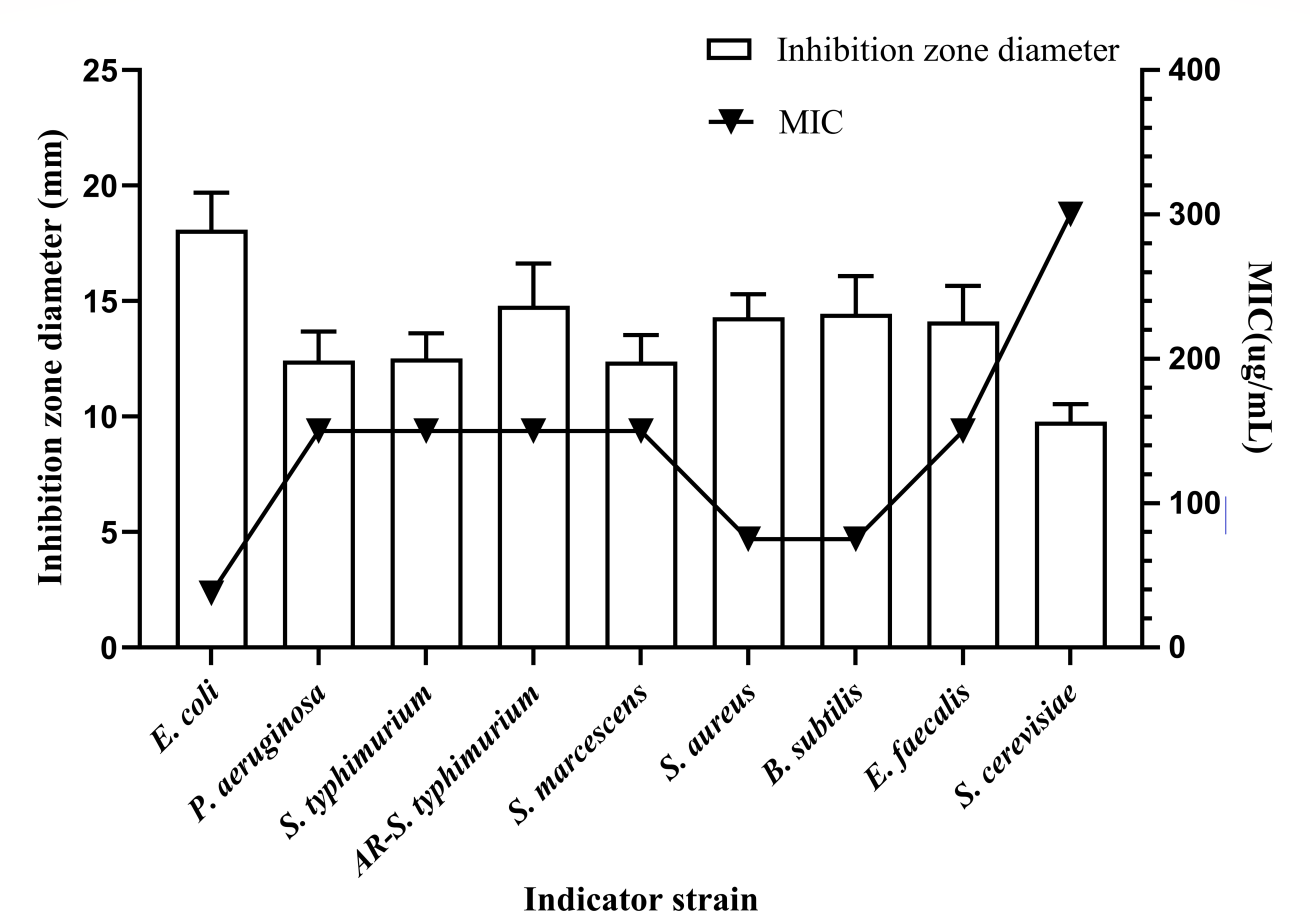
Antimicrobial activity of bacteriocin X24 against indicator strains. X-axis: Indicator strains including G^−^ (*E. coli, P. aeruginosa*, *standard and antibiotic-resistant (AR) S. typhimurium*, *S. marcescens*), G^+^ (*S. aureus*, *E. faecalis, B. subtilis*), and fungi (*S. cerevisiae*). Left y-axis: Inhibition zone diameter (mm). Right y-axis: MIC (μg/mL).

Antimicrobial activity of bacteriocin X24 against indicator strains, including bacteria (G^+^, G^−^) and fungi (e.g., *Saccharomyces cerevisiae*), with inhibition zone diameters (mm) and MIC values (μg/mL) of bacteriocin X24 against indicator strains, is shown in Fig. 4.

As shown in Fig. 4, bacteriocin X24 exhibits significant antibacterial activity, producing inhibition zones ranging from 9.78 ± 0.61 mm to 18.09 ± 1.31 mm against all indicator strains. Complementary MIC determinations further demonstrated potent antimicrobial activity (37.50–300 μg/mL). Inhibition against each indicator strain intensified concomitantly with increasing bacteriocin concentration (Fig. S3). Bacteriocin X24 displayed selective antibacterial activity. It was most potent against *E. coli* (MIC = 37.5 µg/mL), followed by *S. aureus* and *B. subtilis* (MIC = 75 µg/mL). However, it showed limited efficacy against other G^+^ bacteria such as *P. aeruginosa*, standard and antibiotic-resistant *S. typhimurium*, *S. marcescens,* and G^−^ bacteria *E. faecalis* (MIC = 150 µg/mL), and low antifungal activity against *S. cerevisiae* (MIC = 300 µg/mL) (Fig. 4). Notably, bacteriocins also exhibit significant antimicrobial efficacy against antibiotic-resistant *S. typhimurium*, highlighting their potential as novel antimicrobial agents or adjunctive therapeutics against resistant foodborne diseases. These collective results establish bacteriocin X24’s broad-spectrum antimicrobial properties against diverse G^+^ and G^−^ bacteria, as well as fungi.

### Stability characteristics of bacteriocin X24

**Fig 5:**
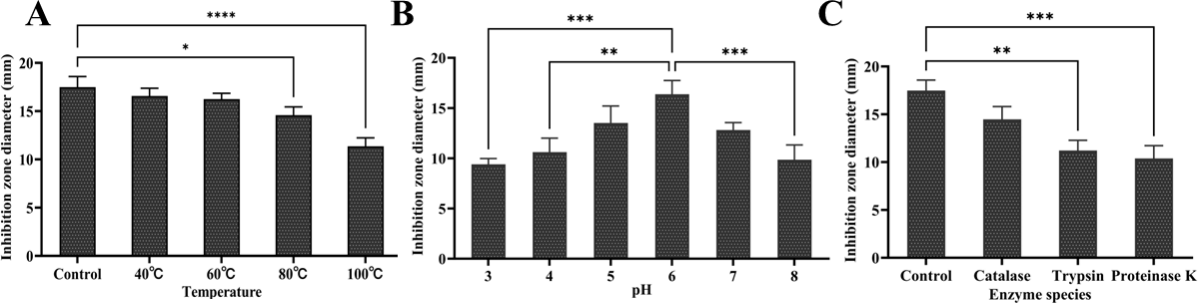
Impact of temperature, pH, and enzymatic conditions on the antimicrobial activity of bacteriocin X24. *P < 0.05, **P < 0.01, ***P < 0.001, ****P < 0.0001. Without labels, indicate no significant difference (ns). (**A**) Temperature. (**B**) pH. (**C**) Enzyme species. Control: no enzyme treatment.

The impact of temperature, pH, and enzymatic conditions on the antimicrobial activity of bacteriocin X24 is presented in Fig. 5.

As shown in Fig. 5A, using untreated samples maintained at 37°C as controls, bacteriocin X24 demonstrates remarkable thermal stability. While its antibacterial activity gradually decreased with increasing temperature, it retained over 90% of activity following 20-min incubations at both 40°C and 60°C. Notably, the bacteriocin maintained 83.40% and 64.95% of its activity even after exposure to 80°C and 100°C for 20 min, respectively.

Figure 5B reveals that bacteriocin X24 reached peak antibacterial activity at pH 6.0 and remained stable within the pH range of 5.0–7.0. However, the activity showed strong pH dependence, decreasing by 42.57% at pH 3.0. Furthermore, significant activity reductions of 35.20% and 39.82% were observed at pH 4.0 and 8.0, respectively.

It could be seen from Fig. 5C that bacteriocin X24 had different degrees of antibacterial activity under the action of different enzymes, among which the antibacterial activity of bacteriocin remained 40.57% when treated by proteinase K. The antibacterial activity of X24 was reduced to 82.82% and 64.18% after treatment with trypsin and catalase, respectively. Proteinase K exerts the most significant effect on the antibacterial activity of the bacteriocin.

Bacteriocin X24 exhibits remarkable thermal stability and pH-dependent antibacterial activity but is protease-sensitive, indicating that its antimicrobial function relies on proteinaceous components.

## DISCUSSION

Consistent with prior studies (27–29), our analysis of *Blattella germanica* gut microbiota revealed Proteobacteria and Firmicutes as the dominant phyla, with Firmicutes accounting for 73.3% of the 30 isolated bacterial strains. Among these, six strains exhibiting LAB characteristics were identified as *Enterococcus* sp., all of which demonstrated bacteriocin production. While *Enterococcus* typically supports digestion, microbial homeostasis, and immune defense in insect guts, certain strains may exhibit opportunistic pathogenicity under compromised host immunity or antibiotic exposure (30).

Notably, all selected LAB strains inhibited *Escherichia coli* and *Staphylococcus aureus*, with strain X24 showing particularly potent antimicrobial activity. Bacteriocins likely exert their effects through growth suppression, metabolic interference, or membrane disruption (31). Like other LAB-derived bacteriocins (32–34), bacteriocin X24 displayed broad-spectrum activity against both G^+^ and G^−^ bacteria, mirroring the properties of a novel bacteriocin from *Lactobacillus plantarum* ZJ5 (35). Strikingly, bacteriocin X24 exhibited enhanced efficacy against antibiotic-resistant *Salmonella typhimurium*, suggesting a potential role in combating antimicrobial resistance (36). Its pronounced activity against G^−^ bacteria may stem from interactions with outer membrane ligands, optimizing bacteriocin binding and potency (37).

The bacteriocin X24 demonstrated exceptional thermal stability (retaining activity at 100°C) and pH adaptability (active at pH 2.0–8.0, optimal at 5.0–7.0), which are critical for food processing and gastrointestinal applications (38). Its stability surpassed previously reported LAB bacteriocins (39), especially in mild acidity. Protease sensitivity (exceeding catalase effects) confirmed its proteinaceous nature, aligning with characteristics of *Enterococcus* F4-9 and RJ-11 bacteriocins (40, 41).

This study identifies the gut bacteria of *Blattella germanica* as a promising source of bacteriocin-producing LAB, laying a foundation for developing novel antimicrobials. Future work should elucidate bacteriocin X24’s mechanism and explore its applications in food preservation and antimicrobial resistance management.

## Data Availability

The raw sequencing data generated in this study have been deposited in the NCBI GEO database under accession numbers PV243577-PV243606.

## SUPPLEMENTAL MATERIAL

Supplement material spectrum.01203-25-s0001.pdf Supplemental methods, and Figures S1 to S3.

ASM does not own the copyrights to Supplemental Material that may be linked to, or accessed through, an article. The authors have granted ASM a non-exclusive, world-wide license to publish the Supplemental Material files. Please contact the corresponding author directly for reuse.
