# Supplementary material for: Characterization and antimicrobial potential of bacteriocin-producing lactic acid bacteria isolated from the gut of Blattella germanica
Source: Microbiol Spectr. 2025 Sep 23;13(11):e01203-25. doi: 10.1128/spectrum.01203-25 (PMC12584616; doi:10.1128/spectrum.01203-25)
Supplement: Supplement material — Supplemental methods, and Figures S1 to S3. [file spectrum.01203-25-s0001.pdf]

Supplementary Material for Characterization and Antimicrobial Potential of Bacteriocin-Producing Lactic Acid Bacteria Isolated from the Gut of *Blattella germanica*

Supplementary Methods

1. Phylogenetic analysis

Phylogenetic analysis was conducted using MrBayes software. The optimal evolutionary model was selected using ModelTest software, and evolutionary distances were calculated based on the GTR+I+G model[1]. Phylogenetic trees were subsequently constructed using Bayesian inference (BI).

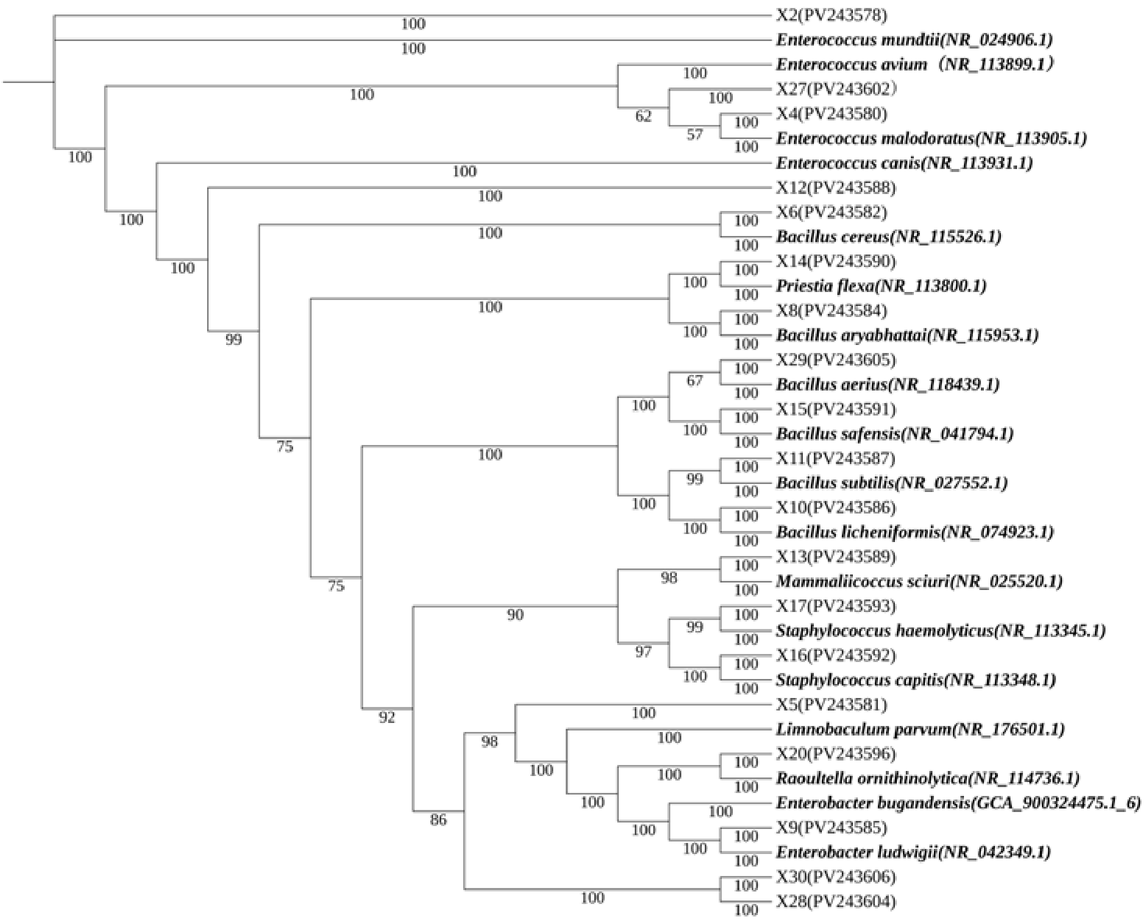

FIG S1 Phylogenetic tree constructed from the gut bacteria 16S rRNA gene sequences of *Blattella germanica* using the Bayesian method [Markov chain Monte Carlo (MCMC) iterations: 1,000,000]. Numbers in brackets indicate the GenBank accession numbers of the sequences. Bootstrap values are shown at the nodes, representing the percentage support for each branch of the evolutionary tree.

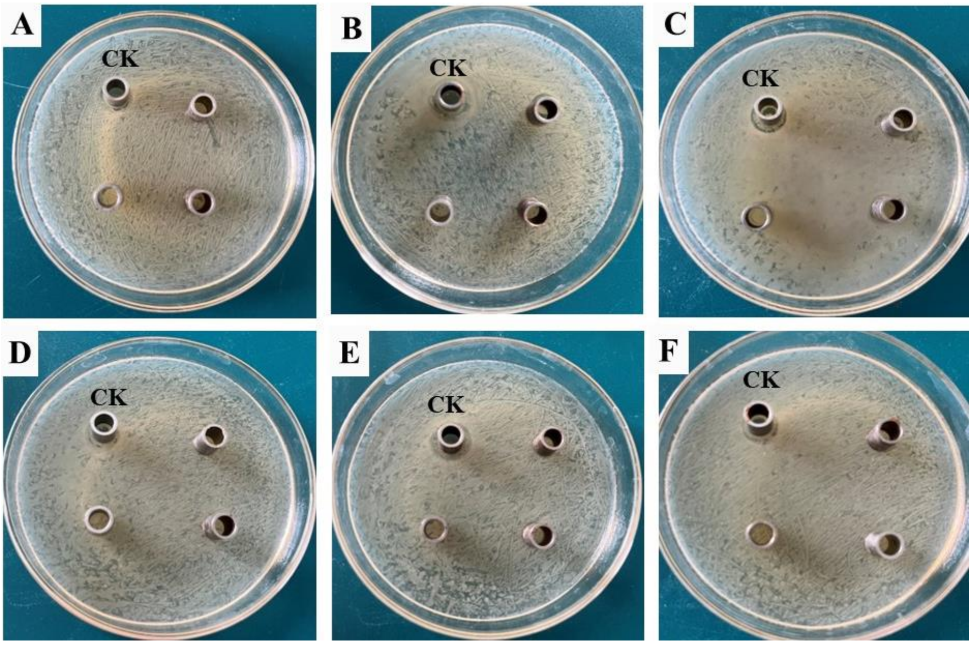

FIG S2 Assay of antibacterial activity of the fermentation supernatant with and without protease (protease K and trypsin). CK: fermented supernatant (with inhibition zone); and three treated experimental replicates: no antibacterial activity (without inhibition zone) was detected after protease treatment; A-F: Fermented supernatant treated with protease of strains X1, X4, X12, X24, X25, and X27 respectively; *Escherichia coli* as indicator strain.

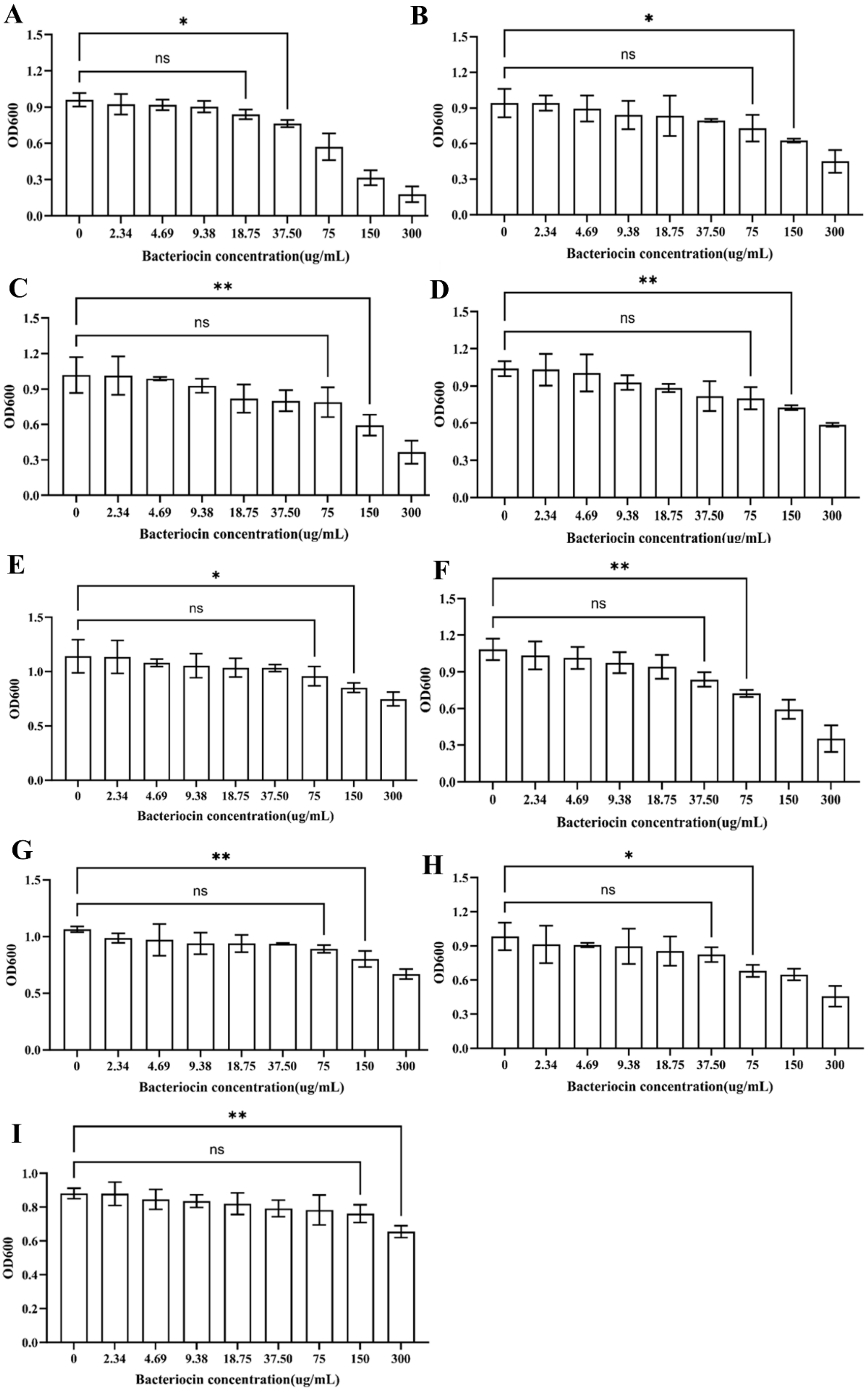
A
*
ns
OD600
1.5
1.2
0.9
0.6
0.3
0.0
0
2.34
4.69
9.38
18.75
37.50
75
150
300
Bacteriocin concentration(ug/mL)
B
*
ns
OD600
Bacteriocin concentration(ug/mL)
C
**
ns
OD600
Bacteriocin concentration(ug/mL)
D
**
ns
OD600
Bacteriocin concentration(ug/mL)
E
*
ns
OD600
1.5
1.0
0.5
0.0
Bacteriocin concentration(ug/mL)
F
**
ns
OD600
Bacteriocin concentration(ug/mL)
G
**
ns
OD600
Bacteriocin concentration(ug/mL)
H
*
ns
OD600
Bacteriocin concentration(ug/mL)
I
**
ns
OD600
1.0
0.8
0.6
0.4
0.2
0.0
Bacteriocin concentration(ug/mL)

FIG S3 Minimum inhibitory concentrations (MICs) of X24 bacteriocin against different indicator strains. A-I: *Escherichia coli*, *Pseudomonas aeruginosa*, *Salmonella*, *drug-resistant Salmonella*, *Serratia marcescens*, *Staphylococcus aureus*, *Enterococcus faecalis*, *Bacillus subtilis*, *Saccharomyces cerevisiae*.

Reference

[1] Wang J, Chen F, Xiao X, Yang X and Xia W (2025). A Comprehensive Protocol for Bayesian Phylogenetic Analysis Using MrBayes: From Sequence Alignment to Model Selection and Phylogenetic Inference. *Bio-protocol* 15(8): e5276. DOI: 10.21769/BioProtoc.5276.
